# Brainstem clinical and neurophysiological involvement in COVID-19

**DOI:** 10.1007/s00415-021-10474-0

**Published:** 2021-03-18

**Authors:** Tommaso Bocci, Gaetano Bulfamante, Laura Campiglio, Silvia Coppola, Monica Falleni, Davide Chiumello, Alberto Priori

**Affiliations:** 1 Clinical Neurology Unit, University of Milan, Milan, Italy; 2 Pathology and Medical Genetics Unit, University of Milan, Milan, Italy; 3 Intensive Care, Anesthesia and Resuscitation Unit, University of Milan, Milan, Italy; 4 ASST Santi Paolo and Carlo and Department of Health Sciences, University of Milan, Milan, Italy; 5 “Aldo Ravelli” Center for Neurotechnology and Experimental Brain Therapeutics, University of Milan, Milan, Italy; 6 Divisione di Neurologia I, Ospedale Universitario San Paolo, Via Antonio di Rudinì 8, 20142 Milan, Italy

Dear Sirs,

Whilst respiratory failure in COVID-19 arises from severe interstitial lung involvement [17], SARS-CoV-2 likely spreads also through the nervous system [3, 6]. Before that SARS-CoV-2 emerged at a global scale [14], other coronaviruses have been proven to invade the brainstem in mice [5] and humans [1, 2, 7]. SARS-Cov-2 might spread cell-to-cell in a prion-like way [3, 8, 11], along the vagus nerve, reaching respiratory centers in the brainstem, possibly adding a neurogenic component to the respiratory failure [11, 15]. To test this hypothesis, we assessed neurophysiologically and clinically the brainstem in patients admitted to the Intensive Care Unit (ICU; time of hospitalization: 10.5 ± 4.8 days, mean ± standard deviation).

The blink reflex (BR) was assessed in 11 severe COVID-19 patients (9 males, mean age 55.2 ± 7.1 years, range 48–70; Fig. 1) [4, 9]. Diagnosis of COVID-19 was confirmed by positive results on a reverse-transcriptase-polymerase-chain-reaction (RT-PCR) assay performed on nasopharyngeal and throat swab, or on lower respiratory tract specimens; each patient suffered from interstitial pneumonia typical of SARS-CoV-2 disease and was intubated at the time of the neurological evaluation. The BR includes two responses: the first is mediated by a disynaptic pathway between the sensory nucleus of the trigeminal nerve (V) in the mid pons and the ipsilateral facial nucleus in the lower pontine tegmentum. The second response (RII) originates through a multi-synaptic circuit in the medulla oblongata [9]. The supraorbital nerve was stimulated through a pair of silver chloride cup electrodes with the cathode over the supraorbital foramen and the anode 2 cm above (constant current stimulation, pulse width 200 µs, inter-trial interval ranging between 25 and 35 s to avoid habituation) [4, 9]. A total of 8 blink reflexes were recorded on each side and data were collected from superimposed traces. Electromyographic signal was captured by surface electrodes and analyzed (band-pass 10 Hz–10 kHz, sampling rate 5 kHz, sensitivity set 200 µV/Div; sweep speed 10 ms/Div). Neurophysiological responses in COVID-19 patients were compared with those from 15 age-matched healthy volunteers and 5 non-COVID ICU intubated patients (Fig. 1). The glabellar and corneal reflexes were also clinically tested; the response to both reflexes was labeled as normal (score = 2), reduced (1) or absent (0).

**Fig. 1:**
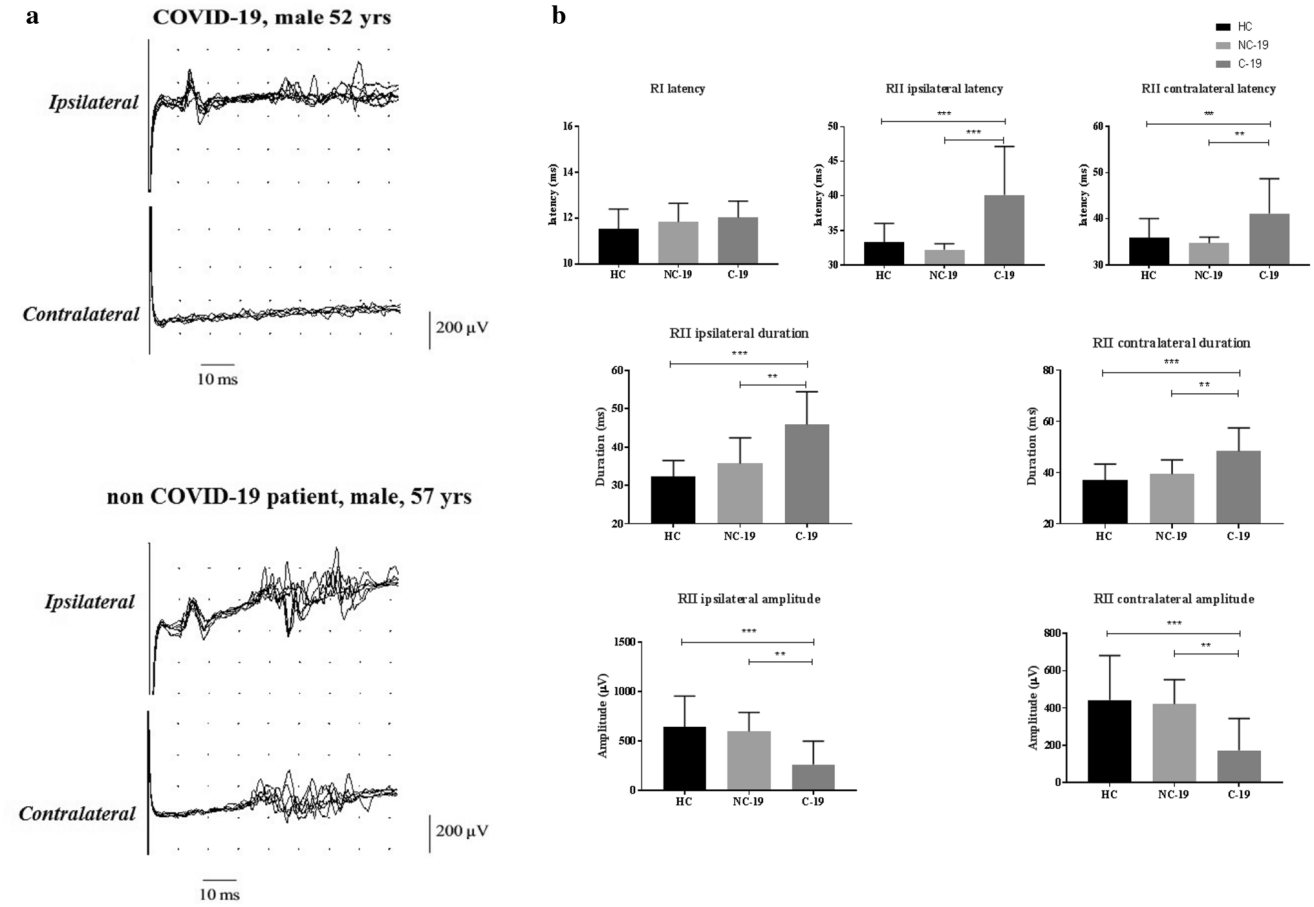
Neurophysiology **a** The blink reflex (BR) in two representative cases: eight superimposed raw traces, recorded in a COVID-19 patient (top) and in in a non-COVID-19 patient (bottom); note that, in the COVID-19 case, ipsilateral RII responses had markedly prolonged latencies and contralateral RII were absent. **b** Histograms showing RI and RII (ipsilateral and contralateral responses) onset latency (top), amplitudes (middle) and duration (bottom), comparing age- and sex-matched healthy controls (HC, black), non-COVID ICU patients (NC-19, gray) and COVID-19 patients (C-19, dark gray). Note that, whereas RI latencies did not differ among groups, both RII latencies and duration (ipsilateral and contralateral) were significantly prolonged in COVID-19 patients. The bulbar RII amplitude was significantly decreased in COVID-19 group. Histograms are mean values, error bars are standard deviation. In the neurophysiological assessment, parametric analyses were used, as all datasets passed the Shapiro–Wilk test for normality (*p* > 0.05). A one-way repeated measures ANOVA was used to compare neurophysiological data among groups (healthy volunteers, COVID-19 and other ICU patients) and the Bonferroni method served as post-hoc comparison (statistical significance set at *p* < 0.017; ***p < 0.0001; **p < 0.01)

At the time of evaluation, patients were awake and in the 60 min before none of them assumed drugs interfering with neuromuscular transmission or depressing the central nervous system.

Whereas all the COVID-19 patients had normal pontine RI latencies (*p* = 0.1), in two of them the RII was absent and in the remaining cases markedly abnormal, both the ipsilateral (latency: *p* < 0.0001; amplitude: *p* < 0.0001; duration: *p* < 0.0001) and the contralateral response (latency: *p* = 0.0014; amplitude: *p* < 0.0001; duration: *p* < 0.0001; see Table 1).

**Table 1:** Data concerning the medullary RII component of the blink reflex (BR)

	Ipsilateral R2			Contralateral R2		
	Latency (ms)	Amplitude (µV)	Duration (ms)	Latency (ms)	Amplitude (µV)	Duration (ms)
Covid-19	40.1 ± 7.0	261.6 ± 235	45.9 ± 8.7	41.2 ± 7.5	172.6 ± 171.2	48.3 ± 9.1
Healthy Controls	33.3 ± 2.7	638.7 ± 316	32.2 ± 4.2	35.9 ± 4.1	443.0 ± 239	37.0 ± 6.3
Non Covid-19 ICU patients	32.2 ± 1.0	597.2 ± 192	35.7 ± 6.7	34.7 ± 1.3	421.5 ± 131	39.4 ± 5.5

Among COVID-19 patients, four had an absent glabellar reflex (score = 0), while the others had a markedly impaired reflex (score = 1). The corneal reflex was present in eight COVID-19 patients out of 11, and reduced in the remaining three. Non-COVID patients showed normal glabellar and corneal reflexes.

Our findings provide the neurophysiological and clinical evidence of SARS-Cov-2-related brainstem involvement in severe Covid-19 patients, especially at the medullary level. Our results agree with recent histopathological data showing a preferential involvement of the lower medulla, without any evidence of intracerebral bleeding or small-vessels thromboses, and confirming the intraneuronal localization of SARS-Cov-2 nucleoprotein [13].

Although SARS-Cov-2-related Guillain-Barré syndrome has been recently reported, mainly of axonal type and with an early involvement of the cranial nerves [16], normal RI latencies and amplitudes rule out this diagnosis in our patients.

From a clinical perspective, the glabellar reflex was impaired more than the corneal. The two reflexes rely on slightly different circuits, targeting the pontine sensory nucleus and the nucleus of the spinal tract of the trigeminal nerve, then projecting to the facial nucleus and reticular formation (RF). However, the amount of fibers reaching the interneuronal network of the medullary reticular formation is lower for the corneal than for the glabellar reflex, probably accounting for the differences we observed [9].

Overall, our results suggest that the brainstem involvement likely contributes to respiratory failure in COVID-19 patients as postulated by Manganelli [12] and Baig [3].

Yet, BR assesses a ponto-medullary circuitry partly involving the reticular formation [9] close to the respiratory nuclei. The reticular formation itself modulates the activity of the respiratory centers [10].

## Data Availability

The corresponding author has full access to data and has the right to publish such data. Data will be available upon reasonable request to the corresponding author.
